# Organic semiconductor heterojunctions: electrode-independent charge injectors for high-performance organic light-emitting diodes

**DOI:** 10.1038/lsa.2016.42

**Published:** 2016-03-11

**Authors:** Yong-Hua Chen, Dong-Ge Ma, Heng-Da Sun, Jiang-Shan Chen, Qing-Xun Guo, Qiang Wang, Yong-Biao Zhao

**Affiliations:** 1 State Key Laboratory of Polymer Physics and Chemistry, Changchun Institute of Applied Chemistry, Chinese Academy of Sciences, Changchun 130022, China; 2 Department of Macromolecular Science and Engineering, School of Engineering, Case Western Reserve University, Cleveland, OH 44106, USA; 3 School of Materials Science and Engineering, Shaanxi Normal University, Xi'an 710062, China; 4 Luminous! Center of Excellence for Semiconductor Lighting and Displays, School of Electrical and Electronic Engineering, Nanyang Technological University, Singapore 639798, Singapore

**Keywords:** charge injection, OLEDs, organic semiconductor heterojunctions

## Abstract

Organic light-emitting diodes (OLEDs) are driven by injected charges from an anode and a cathode. The low and high work function metals are necessary for the effective injection of electrons and holes, respectively. Here, we introduce a fully novel design concept using organic semiconductor heterojunctions (OSHJs) as the charge injectors for achieving highly efficient OLEDs, regardless of the work functions of the electrodes. In contrast to traditional injected charges from the electrodes, the injected charges originate from the OSHJs. The device performance was shown to be significantly improved in efficiency and stability compared to conventional OLEDs. Attractively, the OLEDs based on OSHJs as charge injectors still exhibited an impressive performance when the low work function Al was replaced by air- and chemistry-stable high work function metals, such as Au, Ag, and Cu, as the cathode contact, which has been suggested to be difficult in conventional OLEDs. This concept challenges the conventional design approach for the injection of charges and allows for the realization of practical applications of OLEDs with respect to high efficiency, selectable electrodes, and a long lifetime.

## Introduction

A major issue with organic light-emitting diodes (OLEDs) is that electrons and holes should be effectively injected into the emissive layers^1^. Low energy barriers at the electrode/organic film interface are desirable for efficient charge injection and are generally a prerequisite for high device performance^2,3,4^. The low and high work function metals, therefore, have to be employed in the cathode and anode to facilitate the injection of electrons and holes, respectively^2^. However, this results in drawbacks, including the diffusion of metal ions, such as indium, from a common indium tin oxide (ITO) anode into the emissive layers of OLEDs^5^ and the accumulation of space charges at the interface due to the injected barriers between electrodes and organic semiconductors^2^, which leads to the degradation of device performance over time. Moreover, the low work function metals are very sensitive to moisture and oxygen in the air, which often form detrimental quenching sites near the interface between the emissive layer and the cathode^6^. By using air- and chemistry-stable high work function metals, such as Au, Ag, and Cu, as the cathode, the degradation effect caused by moisture and oxygen in the air can be avoided. Unfortunately, it has been proven that the electrons are very difficult to effectively inject due to their higher injection barrier^2^. Even when an interfacial layer^7,8,9^ or n-type-doped organic layer^10,11,12^ is introduced, the low work function metals also have to be used as the cathode contact to guarantee the effective injection of electrons. Likewise, the anode must also be a high work function metal to realize the effective hole injection, even when inserting an interfacial layer^7,13,14,15^ or p-type-doped organic layer^10,12,16^. This means that the device performance is strongly dependent on the work function of metal electrodes in conventional OLEDs, which is difficult to resolve. More importantly, the instability caused by defects and the high space electric field due to charge accumulation at the interface between electrodes and organics is detrimental to the efficiency and lifetime of OLEDs^17^. This problem is generally also very difficult to control and resolve in the design of conventional OLEDs due to the limitations of the working principle.

It has been shown that charge generation layers (CGLs) in tandem OLEDs can effectively generate charges and realize the injection of charges into respective electroluminescent (EL) units under external electric fields^18,19,20,21,22^. Like metals, CGLs play the important role of electrodes, although they are floated within the devices. Theoretically, CGLs can serve as electrodes to realize the injection of both electrons and holes, but they are completely different from the metal electrodes in conventional OLEDs. In the case of CGLs, the injected charges originate from the generated charges in CGLs, and the injection is directly from the CGLs into the EL units. Therefore, the problems caused by a metal/organic interface could be greatly reduced or eliminated.

Although CGLs are feasible as charge injectors, to the best of our knowledge, there is no direct experimental evidence demonstrating that conventional CGLs widely used in tandem OLEDs can realize the effective charge injection by being inserted on the anode and cathode sides. Recently, we found that organic semiconductor heterojunctions (OSHJs), consisting of a layer of p-type organic semiconductor and a layer of n-type organic semiconductor, can generate large amounts of charges, and they were successfully used as CGLs to fabricate tandem OLEDs with improved power efficiency, showing a better charge generation effect than conventional CGLs^23,24,25,26,27^. More importantly, they can be used as charge injectors to effectively realize the large injection of both electrons and holes via their highly effective charge generation effect, and thus, an electrode-independent high-performance OLED was successfully fabricated.

Here, we report on the novel design concept using OSHJs as charge injectors to realize high-performance OLEDs. Using a C_60_/pentacene OSHJ as an example, we systematically investigate the charge generation and injection in a C_60_/pentacene OSHJ under an external electric field and fabricate OLEDs based on C_60_/pentacene OSHJs as hole injectors and electron injectors, respectively. The resulting OLEDs that are based on this OSHJ as the charge injector show the same or even higher efficiency and stability than conventional OLEDs. Most significantly, the impressive performance can be achieved despite using an air- and chemistry-stable high work function metal, such as Au, Ag, or Cu, as the electric contact, which has been suggested to be very difficult with conventional OLEDs. Our results not only challenge the design concept in OLEDs but also give a wide choice of electrodes, including metals, conductive metal oxides, and polymers, that can be used to fabricate high-performance OLEDs without the need to control their work functions.

## Materials and Methods

### Device fabrication and testing

Devices were grown on cleaned glass substrates pre-coated with a 180-nm-thick layer of ITO with a sheet resistance of 10 Ω per square, as in our previous reports^23,24,25,26,27^. All layers were deposited by thermal evaporation layer by layer without breaking the vacuum (∼5 × 10^−4^ Pa). Film thickness was monitored by frequency counters and calibrated by a Dektak 6 M profiler (Veeco, Karlsruhe, Germany). The overlap between the ITO and Al electrodes was the active emissive area of 4 mm × 4 mm for all devices. Current-voltage-brightness characteristics were recorded using a sourcemeter (Keithley 2400, Cleveland, Ohio, USA) and a multimeter (Keithley 2000, Cleveland, Ohio, USA) with a calibrated silicon photodiode. All devices were encapsulated under an N_2_ atmosphere prior to testing, and all the measurements were carried out in ambient atmosphere.

### Lifetime testing

The lifetime measurements were carried out in a glove box at current densities of 6.25 mA cm^–2^ and 12.5 mA cm^–2^ for both conventional OLEDs and OSHJ-based OLEDs.

The luminance degradation was recorded every hour. The lifetime was extrapolated according to

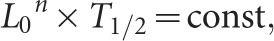


where *L*_0_ is the initial luminance, *T*_1/2_ is the time needed for the luminance to decrease to 50% of the initial value, and *n* is an acceleration exponent. The acceleration factor (*n*) of both OLEDs was estimated to be approximately 1.7.

### Reflectance calculation

The reflectance spectra of Al, Ag, Cu, and Au metal contact electrodes were calculated by the transfer matrix method.

## Results and Discussion

**Figure 1:**
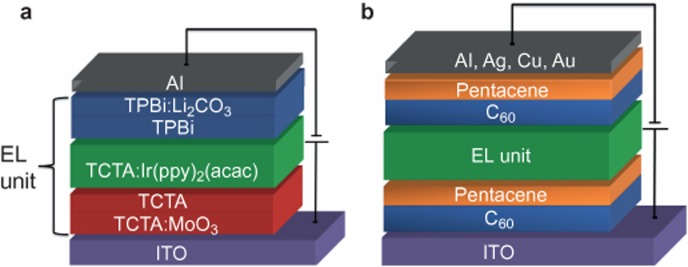
Schematic diagram of the OLEDs used in this study and the device operational mechanism. (**a**) The conventional OLED with a structure of ITO/TCTA:MoO_3_(70 nm)/TCTA(10 nm)/TCTA: Ir(ppy)_2_(acac)(20 nm)/TPBi(10 nm)/TPBi:Li_2_CO_3_(40 nm)/Al(120 nm). (**b**) The organic heterojunction-based OLED with a structure of ITO/C_60_(20 nm)/pentacene(10 nm)/TCTA:MoO_3_(70 nm)/TCTA(10 nm)/TCTA: Ir(ppy)_2_(acac)(20 nm)/TPBi(10 nm)/TPBi:Li_2_CO_3_(40 nm)/C_60_(20 nm)/pentacene(10 nm)/Al(120 nm). We note that the conventional device shown here is under its optimum structure.

The schematic diagrams of the resulting OLEDs without and with C_60_/pentacene OSHJs as the charge injectors are presented in Figure 1a and 1b, respectively. Unlike the conventional OLEDs with metals as charge injectors, a C_60_/pentacene OSHJ is located on each side of the ITO and Al in the studied devices (Figure 1b), where the ITO and Al only play the role of electric contact. The holes and electrons are generated by the charge transfer from pentacene to C_60_, which has been clearly demonstrated in our previous work and by other groups^27,28,29,30,31^. The generated holes and electrons are then extracted and injected into respective EL units upon an external bias and finally lead to the light emission. A common metal organic phosphore of bis(2-phenylpyridine)iridium acetylacetonate (Ir(ppy)_2_(acac)) is introduced and doped into a host of 4,4′,4″-tri(N-carbazolyl)triphenylamine (TCTA) as the emissive layer, which is sandwiched between the hole/exciton-blocking layer of 2,2′,2″-(1,3,5-benzenetriyl) tris-(1-phenyl-1H-benzimidazole) (TPBi) and the electron/exciton-blocking layer of TCTA. A p-type doping layer of TCTA:MoO_3_ and an n-type doping layer of TPBi:Li_2_CO_3_ are employed as the hole-injection/transporting layer and electron-injection/transporting layer, respectively.

**Figure 2:**
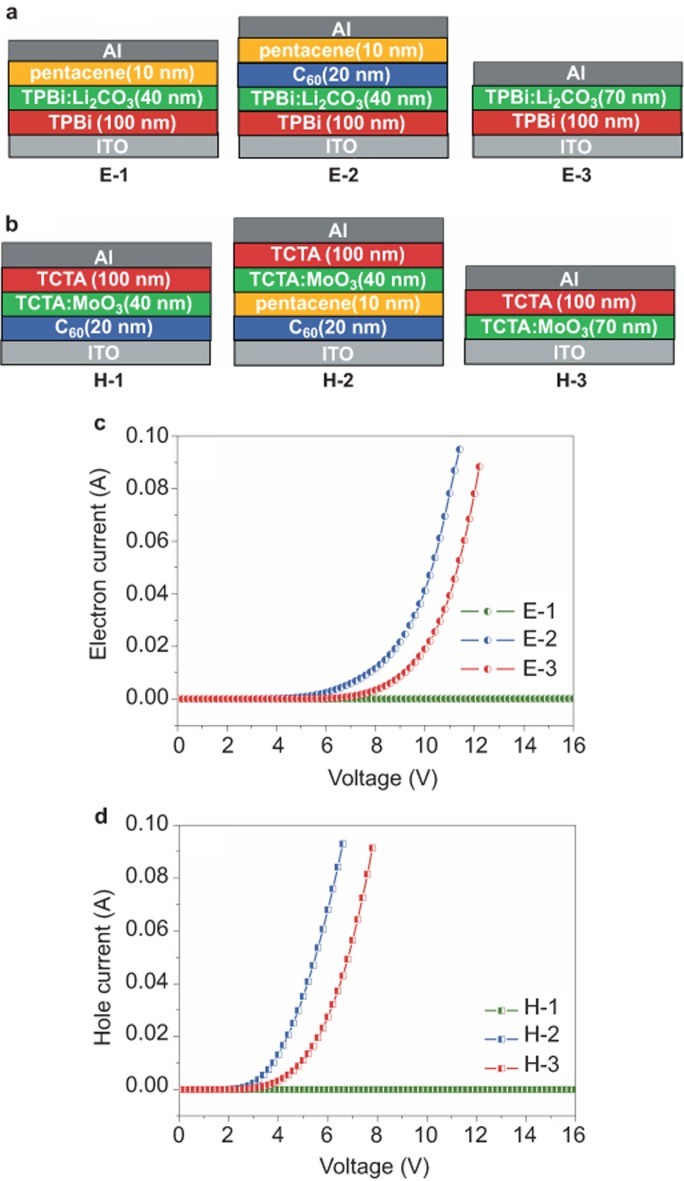
Device structures and *J-V* characteristics of electron- and hole-only devices. (**a**) Electron-only devices. (**b**) Hole-only devices. (**c**) *J-V* characteristics of the electron-only devices without (E-1 and E-3) and with (E-2) C_60_/pentacene OSHJs. (**d**) *J-V* characteristics of the electron-only devices without (H-1, H-3) and with (H-2) C_60_/pentacene OSHJs.

To clarify that the injected electrons and holes originate from the generated charges in the C_60_/pentacene OSHJ rather than from those injected from the external electrodes (ITO and Al) in our C_60_/pentacene OSHJ-based OLEDs, electron-only (Figure 2a) and hole-only (Figure 2b) devices are fabricated. Figure 2c and 2d shows the current density-voltage (*J-V*) characteristics of these devices. It is clearly observed that the electron-only device of ITO/TPBi (100 nm)/TPBi:Li_2_CO_3_ (40 nm)/pentacene (10 nm)/Al (device E-1) and the hole-only device of ITO/C_60_ (20 nm)/TCTA:MoO_3_(40 nm)/TCTA (100 nm)/Al (device H-1) show hardly any current flow within the devices despite the high bias voltage of 20 V between the ITO positive bias and the Al negative bias (Figure 2c and 2d). The extremely low currents in these two devices should be attributed to the large injection and transport barrier between the ITO (∼4.7 eV) and electron-transporting organic TPBi (highest occupied molecular orbital (HOMO) ∼6.2 eV)^32^ and C_60_ (HOMO ∼6.2 eV)^33^ for holes and between the Al (∼4.3 eV) and hole-transporting organic TCTA (lowest unoccupied molecular orbital (LUMO)∼ 2.7 eV)^32^ and pentacene (LUMO ∼3.0 eV)^33^ for electrons. This demonstrates that the hole injection from ITO/C_60_ and the electron injection from Al/pentacene are impossible. However, when inserting a C_60_ layer in device E-1 and a pentacene layer in device H-1, the electron-only device of ITO/TPBi (100 nm)/TPBi:Li_2_CO_3_ (40 nm)/C_60_ (20 nm)/pentacene (10 nm)/Al (device E-2) and the hole-only device of ITO/C_60_ (20 nm)/pentacene (10 nm)/TCTA:MoO_3_ (40 nm)/TCTA (100 nm)/Al) (device H-2) show very large electron and hole currents, respectively. Because no external charge carriers can be injected into both devices from ITO and Al electrodes, as demonstrated in devices H-1 and E-1, it is definitively proven that the large current is obviously due to the generated charges in the C_60_/pentacene OSHJ under the external electric-field induction. This strongly indicates that the C_60_/pentacene OSHJ is an extremely effective charge injector that can generate electrons, and that holes are also largely injected.

To further evaluate the large current injection characteristics of the C_60_/pentacene OSHJ, we fabricated the electron-only device of ITO/TPBi (100 nm)/TPBi:Li_2_CO_3_ (70 nm)/Al (device E-3) and the hole-only device of ITO/TCTA:MoO_3_ (70 nm)/TCTA (100 nm)/Al (device H-3) for comparison. For the purpose of confirming the same electric field intensity, the total thickness of the E-3 and H-3 devices was designed to be the same as that of the E-2 and H-2 devices. In devices E-3 and H-3, the electron injection from the Al cathode and the hole injection from the ITO anode are extremely effective due to the introduction of an n-type doping layer TPBi:Li_2_CO_3_ and a p-type doping layer TCTA:MoO_3_, which are widely used in conventional OLEDs to enhance charge carrier injection^10,12^. As shown in Figure 2, the large electron current and hole current are well realized in devices E-3 and H-3. However, the current in devices E-3 and H-3 is still less than that in devices E-2 and H-2 based on C_60_/pentacene OSHJs as the charge injectors, further demonstrating the validity of C_60_/pentacene OSHJs as charge injectors.

Because a C_60_/pentacene OSHJ is widely used as the active medium in organic solar cells due to its wide absorption in visible wavelength^33^, it may be harmful to OLEDs. However, as shown in Supplementary Fig. S1, where the transmission spectra of the present C_60_ (20 nm)/pentacene (10 nm) organic heterojunction film (the optimized thickness in this work) deposited on glass is given, it is clearly observed that its transmission reaches over 73% in the wavelength range of 400–800 nm and up to 84% at the 520 nm green wavelength, corresponding to the emission of Ir(ppy)_2_(acac). This demonstrates that the proper thickness of the C_60_/pentacene heterojunction possesses adequate transparency across the visible range for the effective light output, meeting the demand of OLED emission.

**Figure 3:**
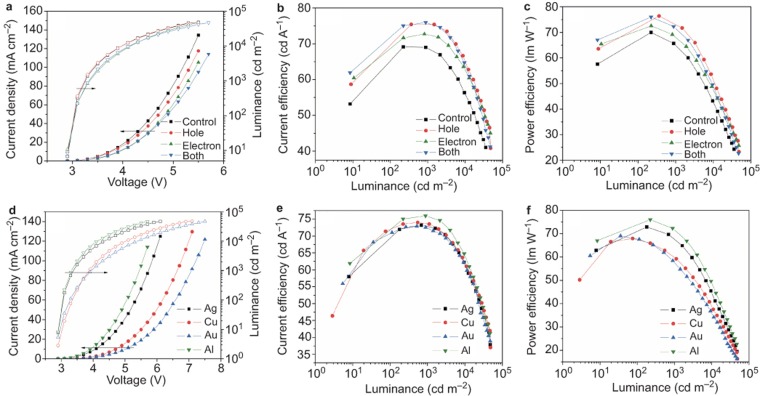
Electroluminescent performances of the conventional OLED and OLEDs with C_60_/pentacene OSHJs as charge injectors. (**a**) *J-V-L* characteristics, (**b**) current efficiency as a function of current density characteristics, and (**c**) power efficiency as a function of current density characteristics of conventional OLED and OLEDs with OSHJs as hole injectors (hole): ITO/C_60_(20 nm)/pentacene(10 nm)/TCTA:MoO_3_(70 nm)/TCTA(10 nm)/TCTA: Ir(ppy)_2_(acac)(20 nm)/TPBi(10 nm)/TPBi:Li_2_CO_3_(40 nm)/Al(120 nm), as electron injectors (electron): ITO/TCTA:MoO_3_(70 nm)/TCTA(10 nm)/TCTA: Ir(ppy)_2_(acac)(20 nm)/TPBi(10 nm)/TPBi:Li_2_CO_3_(40 nm)/C_60_(20 nm)/pentacene(10 nm)/Al(120 nm), and as both hole and electron injectors (both): ITO/C_60_(20 nm)/pentacene(10 nm)/TCTA:MoO_3_(70 nm)/TCTA(10 nm)/TCTA: Ir(ppy)_2_(acac)(20 nm)/TPBi(10 nm)/TPBi:Li_2_CO_3_(40 nm)/C_60_(20 nm)/pentacene(10 nm)/Al(120 nm). (**d**) *J-V-L* characteristics, (**e**) current efficiency as a function of current density characteristics, and (**f**) power efficiency as a function of current density characteristics of OLEDs with OSHJs as both hole and electron injectors for Au, Ag, Cu, and Al metal contact electrodes: ITO/C_60_(20 nm)/pentacene(10 nm)/TCTA:MoO_3_(70 nm)/TCTA(10 nm)/TCTA: Ir(ppy)_2_(acac)(20 nm)/TPBi(10 nm)/TPBi:Li_2_CO_3_(40 nm)/C_60_(20 nm)/pentacene(10 nm)/Au, Ag, Cu or Al (120 nm).

In light of these results showing that C_60_/pentacene OSHJs as charge injectors meet the optical and electrical requirements, as shown in Figure 1, we fabricated green-emission OLEDs with C_60_/pentacene OSHJs as the hole injector and as the electron injector, respectively. Figure 3a displays the current density-luminance-voltage (*J-L-V*) characteristics of OLEDs with a C_60_/pentacene OSHJ on the side of the ITO, the Al and both and those of conventional OLEDs. It can be observed that the OLEDs with OSHJs as the charge injectors show higher EL efficiency than conventional OLEDs. The maximum current efficiency and power efficiency arrive at 75.9 cd A^–1^ and 76.0 lm W^−1^, respectively, and remain at 75.6 cd A^−1^ and 72.1 lm W^−1^ at 1000 cd m^−2^ luminance in OLEDs with C_60_/pentacene OSHJs on both sides of the ITO and Al, and these values are higher than those in conventional OLEDs (shown in Figure 3b and 3c), indicating the highly efficient charge injection property of C_60_/pentacene OSHJs as charge injectors, which is superior to the charge injection directly from electrodes. Moreover, the OSHJ-based devices also work at low current density, indicating more balanced charge transport and recombination in the devices. As shown in Figure 3a, the driving voltage of the OSHJ-based devices does not increase with an increase in the device thickness. The series resistance of the devices, therefore, was analyzed in both the Ohmic region and the turn-on region (Supplementary Fig. S2). As we can see, the devices show the series resistance up to 10^6^ Ω cm^2^ in the Ohmic region (note that there is no light emission in the Ohmic region), and the series resistance of the device with two OSHJs against electrodes (Both) is two times higher than that of the control device (Supplementary Fig. S2a). However, the series resistance is significantly reduced to approximately 11 Ω cm^2^ while the devices are on. Moreover, all four devices exhibit a little variation in the series resistance, which further demonstrates the high conductivity of the OSHJ during operation.

To further understand the function of the OSHJ in our devices and whether it acts as charge generation, the effect of reducing or increasing the thickness of the C_60_ layer close to the anode and the pentacene layer close to the cathode on device performance was investigated. Supplementary Fig. S3 shows the EL performances of the OLEDs using C_60_/pentacene OSHJs as charge injectors with a change to either the C_60_ thickness (Supplementary Fig. S3a–S3c) or the pentacene thickness (Supplementary Fig. S3d–S3f). From current *J-V-L* curves (Supplementary Fig. S3a), we can clearly see that the current density exhibited a slight variation during the whole driving voltage when the thickness of the C_60_ layer was increased or reduced. In detail, the driving voltages were first reduced a little and then increased with an increase in the thickness of the C_60_ layer. Accordingly, the current efficiency and power efficiency were first increased and then reduced. It should be noted that the performance of devices with thicker C_60_ layers (e.g., 30 or 40 nm) was better than that of devices with thinner C_60_ layers (e.g., 10 nm), which strongly demonstrates that the current originates from the charge carriers generated at the C_60_/pentacene OSHJ interface rather than being injected from the ITO anode. In the same way, we changed the thickness of the pentacene close to the cathode to see how the device performance changes. It can be observed from the *J-V-L* curves shown in Supplementary Fig. S3d that the current density also showed a little variation, and the device with a thicker pentacene layer (15 nm) showed higher current density than that with a thinner pentacene layer (5 nm). Therefore, we can draw the conclusion that the injected charges in OSHJ-based devices indeed originate from the generated charges rather than the charge injection from external electrodes.

More attractively, due to the injected charges originating from the C_60_/pentacene OSHJ, the metal electrodes used here only function to offer an electric field rather than as the contributors of the charges. Therefore, the air- and chemistry-stable high work function metals can also be used as the cathode instead of Al. To demonstrate the versatility of stable metals as electrodes in OSHJ-based OLEDs, we fabricated the OLEDs with C_60_/pentacene OSHJ charge injectors and high work function metals of Au (∼5.1 eV), Ag (∼4.4 eV), and Cu (∼4.7 eV) to replace the low work function metal Al as the cathode contact. As shown in Figure 3d–3f, although the current density and luminance at the same voltage show certain variations, it is impressive that approximately the same efficiency can be obtained and the maximum current efficiency and power efficiency can reach 73.2 cd A^−1^ and 72.9 lm W^−1^ for the Ag device, 74 cd A^−1^ and 68 lm W^−1^ for the Cu device, and 72.9 cd A^−1^ and 69.1 lm W^−1^ for the Au device, showing an electrode-independent charge injection property. This further indicates that the injected charges in OSHJ-based devices indeed originate from the generated charges. We also compared the devices without a C_60_/pentacene OSHJ but with Au, Ag, and Cu electrodes. The device performance is shown in Supplementary Fig. S4. It can be observed that the device performance was much poorer without the C_60_/pentacene OSHJ. In detail, all three devices with Ag, Cu, and Au only showed higher driving voltage and lower current efficiency and power efficiency (Supplementary Fig. S4) compared to those of their corresponding device with the C_60_/pentacene OSHJ (Figure 3d–3f). This strongly demonstrates that the C_60_/pentacene OSHJ layers significantly reduce the electrode dependence in our devices. Although the current density varied with different metal electrodes with different work functions in our OSHJ-based devices, we can clearly see that the charge balance was achieved in all devices, which is evident in the similar current efficiency and power efficiency. This means that the electron injection and hole injection in our OSHJ-based devices increase or decrease at the same time, leading to a balanced charge injection in all OSHJ-based devices and indicating that the charge balance is independent of the metal electrodes with different work functions. We can also see that the Cu and Au devices showed lower luminance than the Al and Ag devices at the same operational voltages (Figure 3d). This could be attributed to the different reflectance characteristics of these metals because Ag, Cu, Au, and Al were used as the top reflective electrodes. As shown in Supplementary Fig. S5, the reflectance spectra of these metals were measured and simulated. Al and Ag exhibited excellent reflective properties over the whole visible spectrum, while Cu and Au showed a remarkable decrease in reflectance below 650 nm in both the experiment and the simulation. On the basis of these results, it appears that all of the conductive materials, including metals, conductive metal oxides, and polymers, can be used as electrodes in OLEDs without the need for control of their work functions due to the excellent charge injection functionality of OSHJs as charge injectors. Table 1 summaries the EL performances of OLEDs with C_60_/pentacene OSHJ charge injectors and conventional OLEDs.

Nevertheless, if the pentacene is replaced by another p-type organic material, such as *N,N′*- Di(1-naphthyl)-*N,N′*-diphenyl-(1,1′-biphenyl)-4,4′-diamine (NPB) or TCTA, for the OSHJ injector assembly, the fabricated OLEDs then show higher working voltages, and the power efficiency is greatly reduced (Supplementary Fig. S6).

**Figure 4:**
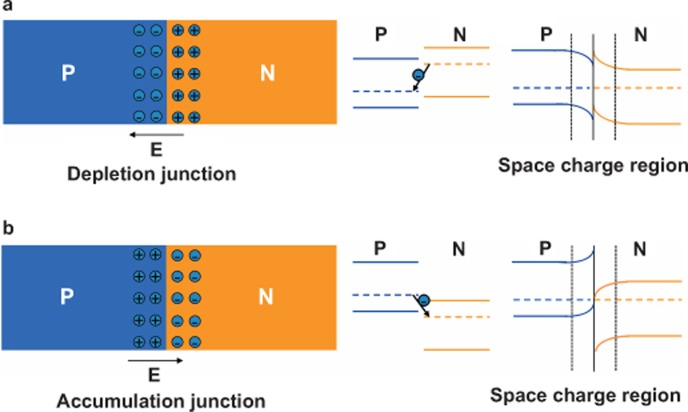
Junction types of semiconductor heterojunctions and their energy levels. (**a**) Depletion junction and (**b**) accumulation junction.

As demonstrated previously^24,27^, the relative energy levels of both semiconductor components play an important role in the construction of effective organic heterojunctions, which directly determine the charge generation and transport. It is well known that for an isotype semiconductor heterojunctions, the energy-band profiles should follow the Anderson rule^34^, which has been extensively adopted in inorganic semiconductor heterojunctions. The most familiar heterojunction type in inorganic semiconductors is the depletion mode. In this type of heterojunction, as shown in Figure 4a, a depletion layer is formed on either side of the interface, and the space charges of these layers are opposite and equal in magnitude, e.g., the positive charges accumulate on the side of the n-type and the negative charges accumulate on the side of the p-type in the depletion region. In this case, these charges in the depletion region are immovable, meaning a high resistance region. To deplete these charges within the space charge region, therefore, an external positive voltage has to be applied to overcome the build-in voltage, which is the opposite of the external voltage. On the other hand, if the Fermi level of the p-type side is higher than that of the n-type side in this heterojunction, the positive and negative charges will accumulate on the sides of p-type and n-type semiconductors, respectively, to form the space charge region. We call this case a junction for the accumulation mode, as shown in Figure 4b. In this case, the build-in voltage is directed from the p-type region to the n-type region, which is the same as the external electric field. More importantly, the accumulation charges within the space charge region are movable. The accumulation of high-density free charge carriers results in the semiconductor heterojunction generally exhibiting a high conductance property along the junction direction. This not only is very helpful for the extraction of charges in the junction region under an external electric field but also will significantly reduce the voltage drop in the junction. Obviously, it can be concluded that the formation of an accumulation junction will be especially beneficial for the operation of semiconductor heterojunctions as CGLs and injectors to supply charges without extra voltage.

Because the dielectric constant of organic semiconductors is usually low, and the non-covalent electronic interactions between organic semiconductors are weak compared to inorganic semiconductors, organic semiconductors construct not only the depletion-type junction but also the accumulation-type junction. This is a special property of OSHJs with respect to inorganic semiconductor heterojunction, which greatly increases the functionality of OSHJs in organic optoelectronic devices^35^. For a heterojunction consisting of C_60_ and pentacene organic semiconductors, the accumulation-type junction is well formed due to the high Fermi level of pentacene compared to that of C_60_ at the interface where the electrons are accumulated in C_60_ and the holes in pentacene^27^. Therefore, as expected, the C_60_/pentacene heterojunction as a charge injector supplies many more free charge carriers and greatly reduces the voltage drop in the heterojunction due to its high conductance space charge region, leading to higher device performance. A recent detailed study on the interfacial electronic structure of buffer-modified C_60_/pentacene heterojunctions by ultraviolet photoelectron spectroscopy and inverse photoemission spectroscopy also demonstrated the properties of highly effective charge generation, transport, and injection in this heterojunction as CGL due to its favorable energy level alignment at the C_60_/pentacene interface^36^, which further supports our results. On the contrary, the C_60_/NPB (or TCTA) heterojunction then forms a depletion-type junction due to the high Fermi level of C_60_ compared to that of NPB (or TCTA)^37^. Although as a charge injector it also realizes approximately the same current density, the power efficiency is greatly reduced due to the need for a large voltage drop in the depletion junction. Our results clearly demonstrate the importance of choice in organic semiconductors to construct the effective heterojunction as charge injectors for high-performance OLEDs.

**Figure 5:**
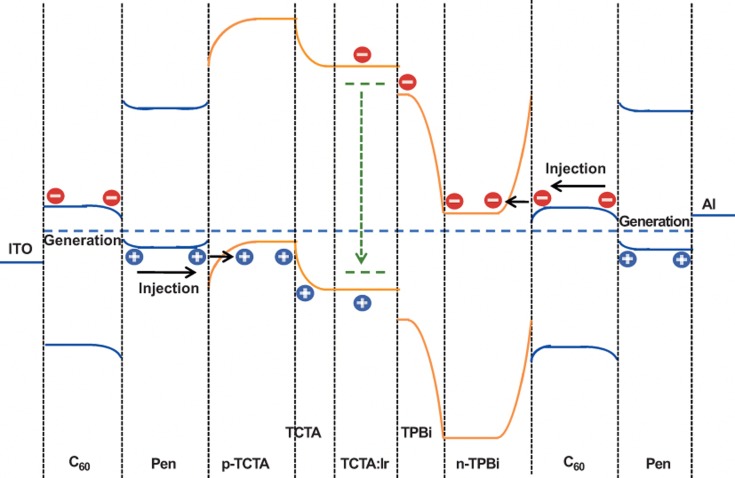
Total energy level diagram of an OLED with a C_60_/pentacene heterojunction as a charge injector at thermal equilibrium and its EL processes.

To elucidate the working processes and the performance-improving mechanism of the OLEDs based on OSHJs as the charge injectors, the total energy level diagram of the devices with a C_60_/pentacene heterojunction as the charge injector at thermal equilibrium is depicted in Figure 5. As previously described, the electrons and holes accumulate on the n-type C_60_ and p-type pentacene, respectively, in the vicinity of the C_60_/pentacene interface. When an external electric field is applied to the ITO and Al electrodes, the accumulated holes at the interface of the C_60_/pentacene are injected into the emissive layer across the hole-transporting layers, and the accumulated electrons move toward the ITO. Simultaneously, the accumulated electrons at the interface of C_60_/pentacene on the Al electrode side are injected into the emissive layer across the electron-transporting layers, and the accumulated holes move toward the Al. Then, the holes and electrons are injected into the emissive layer to form excitons that subsequently emit light upon recombination. Clearly, the injected holes into the emissive layer result from the electron extraction from the pentacene HOMO through the C_60_ LUMO and then into the ITO, instead of a hole transit from the ITO through the C_60_ HOMO, while the injected electrons into the emissive layer result from the hole extraction from the pentacene HOMO through the C_60_ LUMO and then into the Al, instead of an electron transit from the Al through the pentacene LUMO. The injection manner of electrons and holes in the devices based on heterojunctions as the charge injectors is obviously different from that in conventional devices, placing the charge injection far from the problematic electrode interfaces. More importantly, as proven above, the generated charges in OSHJs are determined by the electric field on the heterojunction but are not related to the work function of the electrode metals used. This means that the generated holes on the side of the anode should be approximately equal to the generated electrons on the side of the cathode. As a result, a more balanced hole-electron recombination is realized. The improved balance in the generated charge carriers also prevents an excess of charge from accumulating, whereas the redistribution of the electric field on OSHJs in the devices also reduces the electric field intensity in the emissive region, greatly suppressing the quenching effect of the local field on the emissive excitons. All of these superior properties shown in OLEDs with OSHJs as charge injectors guarantee high efficiency and a long lifetime.

As expected, the fabricated OLEDs with C_60_/pentacene OSHJs as the charge injectors exhibited a longer lifetime than the conventional OLEDs (Supplementary Fig. S7). The lifetime measurement of the devices is made in a glove box at a constant driving current density of 6.25 mA cm^−2^ (4000 cd m^−2^). The half lifetimes arrive at 60 and 47 h for the OSHJ-based devices and the conventional devices, respectively. By using a lifetime acceleration factor of 1.7 power, the lifetimes at an initial luminance of 100 cd m^−2^ are estimated to be approximately 32 000 h for conventional devices and 40 000 h for OSHJ-based devices. The longer lifetime improvement in the OSHJ-based devices should be attributed to the reduced charge quenching caused by the interface defects and the accumulation of space charges between electrodes and organics, which have been suggested to be degradation mechanisms in conventional OLEDs.

## Conclusions

In summary, we demonstrated a fully novel design concept for charge carrier injection by using OSHJs instead of metal electrodes as charge injectors in OLEDs. It can be observed that the charge carriers for light emission are injected from the OSHJs and not from the metal electrodes, challenging the design of charge carrier injection in conventional OLEDs. This has advantages in that the instability caused by defects and the high space electric field due to charge accumulation at the interface between electrodes and organics in conventional OLEDs are greatly reduced or eliminated, and the charge carrier balance is also significantly improved due to the generation of the same electrons and holes at the both sides of electrodes, thus improving the efficiency and stability of fabricated OLEDs. More attractively, the charge carrier injection from the OSHJ is only dependent on the electric field on the heterojunction, and the metal electrodes here only provide an electric field. Therefore, the OSHJs-based OLEDs still achieve excellent EL performance even though they use air- and chemistry-stable high work function metals, such as Au, Ag, and Cu, as contact electrodes, which is generally very difficult in conventional OLEDs. Because the novel charge injection architecture we created is based on a fundamental physical understanding of semiconductor heterojunction theory, OSHJs as charge injectors should be generally applicable to a wide range of phosphorescent and fluorescent devices and different colored devices, including white devices. It is believed that our findings offer an unprecedented versatility and a solid theoretical basis in the design of OSHJs, thus greatly facilitating the further improvement in OLED performance for practical applications, which will ideally inspire further work.

## Figures and Tables

**Table 1:** Summary of EL performances for different devices.

Device type	V_T_^a^ (V)	*η*_cd,max_^b^ (cd A^−1^)	*η*_cd,1000_^c^ (cd A^−1^)	*η*_p,max_^d^ (lm W^−1^)	*η*_p,1000_^e^ (lm W^−1^)
Control	2.9	69.1	68.9	70	65.6
Hole	2.9	75.4	75.3	76.4	73.6
Electron	2.9	72.7	72.2	72.6	68.9
Both	2.9	75.9	75.6	76	72.1
Ag	2.9	73.2	72.8	72.9	67.6
Cu	2.9	74	73.5	68	59.2
Au	2.9	72.9	72	69.1	57.8

a V_T_ is the turn-on voltage defined at the lowest luminance higher than 1 cd m^−2^.

b *η*_cd,max_ is the maximum current efficiency.

c *η*_cd,1000_ is the current efficiency at 1000 cd m^−2^.

d *η*_p,max_ is the maximum power efficiency.

e *η*_p,1000_ is the power efficiency at 1000 cd m^−2^.
