# Supplementary material for: Organic semiconductor heterojunctions: electrode-independent charge injectors for high-performance organic light-emitting diodes
Source: Light Sci Appl. 2016 Mar 11;5(3):e16042–. doi: 10.1038/lsa.2016.42 (PMC6059893; doi:10.1038/lsa.2016.42)
Supplement: Supplementary information [file lsa201642x1.docx]

# Supplementary Information for

**Organic semiconductor heterojunctions: electrode independent charge injectors for high-performance organic light-emitting diodes**

Yonghua Chen1,2, Dongge Ma1,*, Hengda Sun1, Jiangshan Chen1, Qingxun Guo1, Qiang Wang,3

Yongbiao Zhao4

1State Key Laboratory of Polymer Physics and Chemistry, Changchun Institute of Applied Chemistry, Chinese Academy of Sciences, Changchun 130022, China

2Department of Macromolecular Science and Engineering, School of Engineering, Case Western Reserve University, Cleveland, OH 44106, USA

3School of Materials Science and Engineering, Shaanxi Normal University, Xi'an 710062, China 4Luminous! Center of Excellence for Semiconductor Lighting and Displays, School of Electrical and Electronic Engineering, Nanyang Technological University, 50 Nanyang Avenue, Singapore

639798, Singapore

*e-mail: mdg1014@ciac.ac.cn


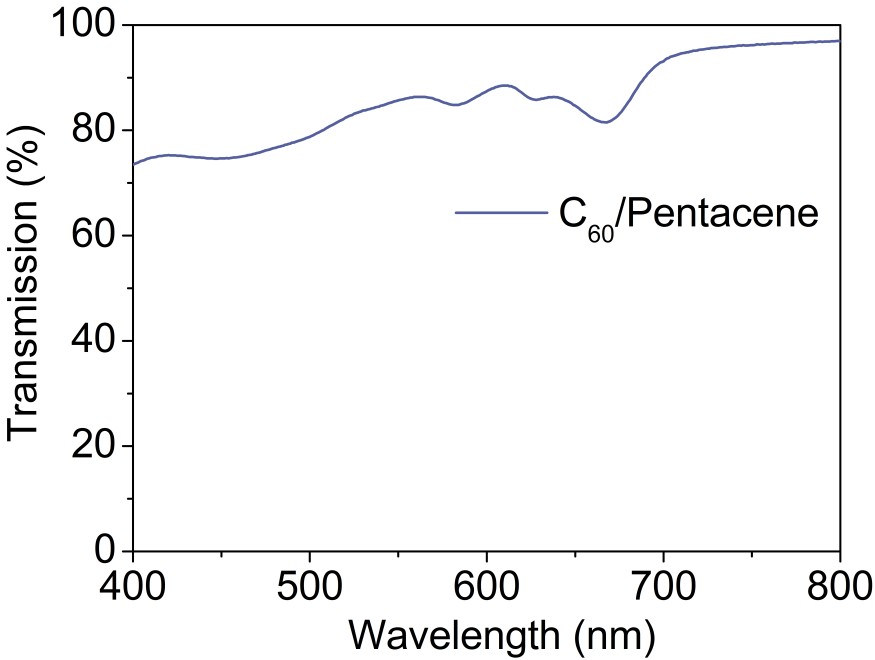


## Figure S1.Transmission spectra of the present C60 (20 nm)/pentacene (10 nm) OSHJ film deposited on glass.


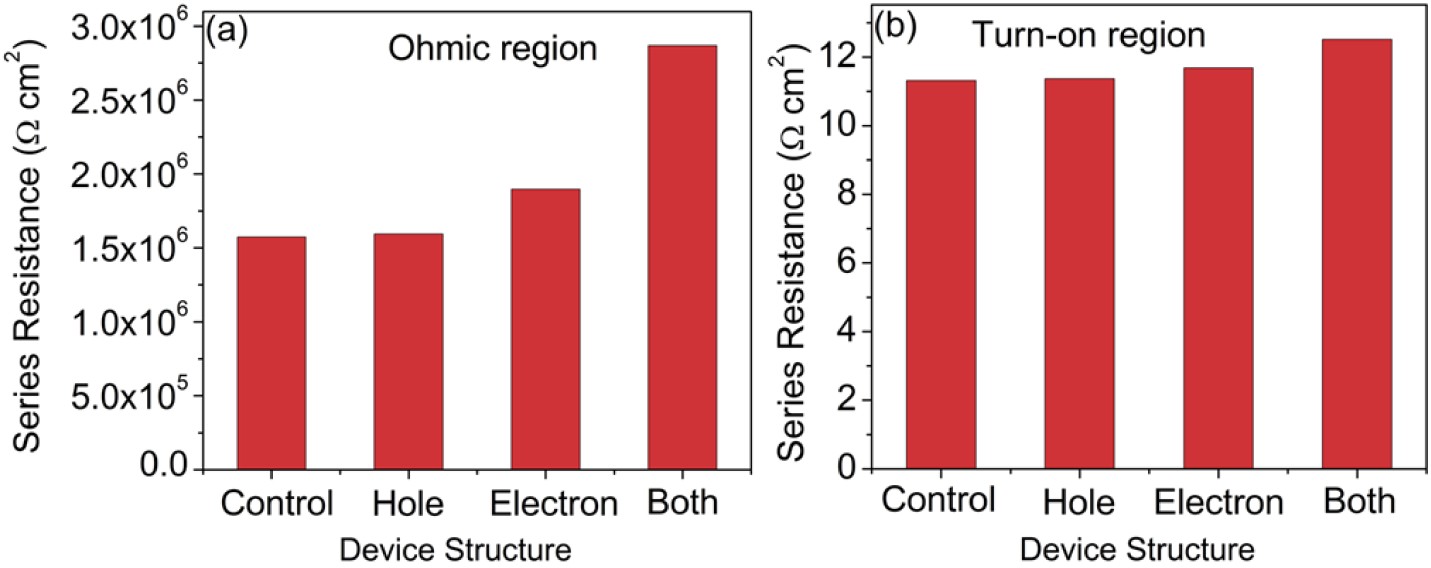


**Figure S2. The series resistance of devices in Ohmic region (a) and turn-on region (b).**

**
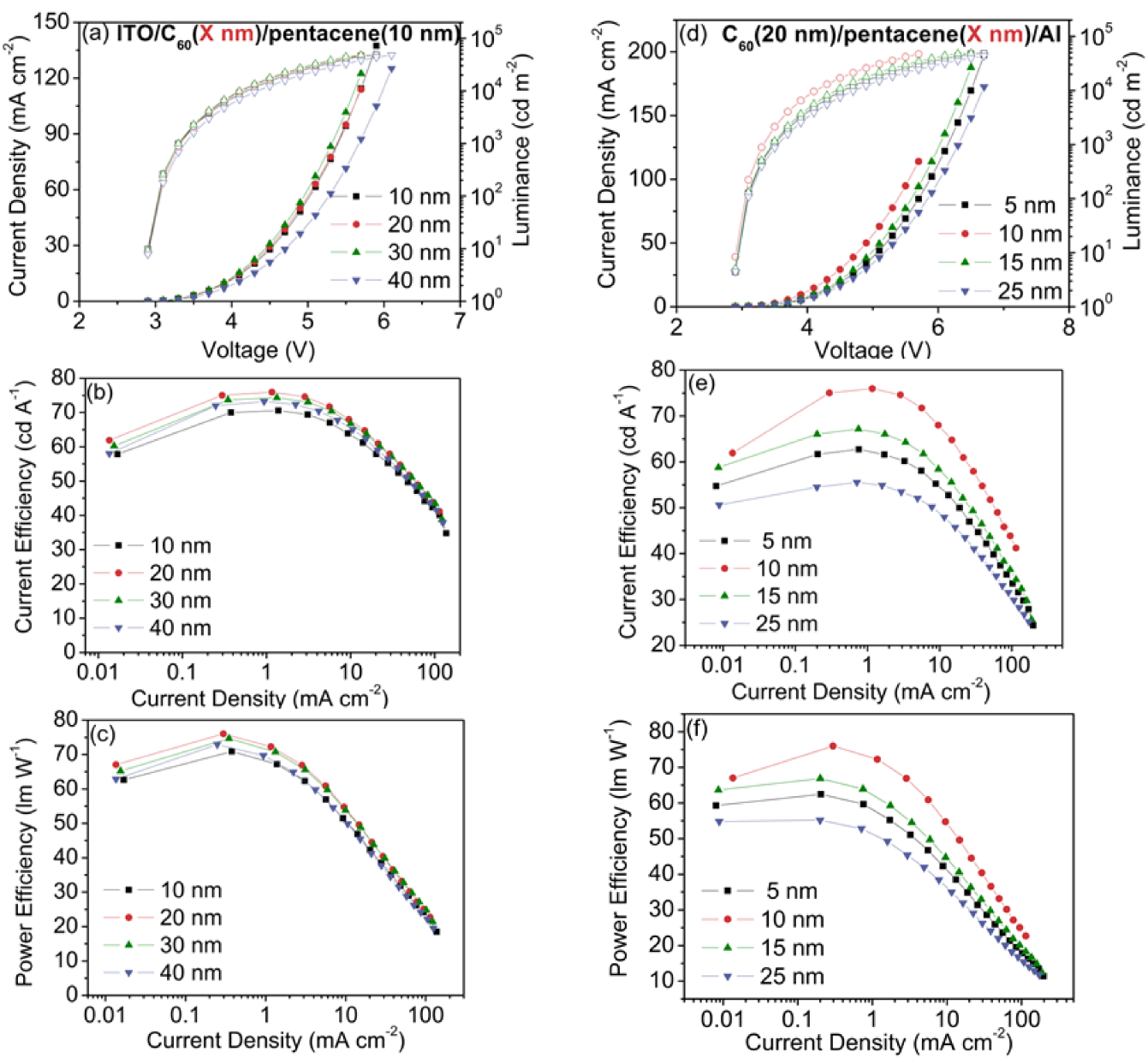
**

**Figure S3. EL performances of the OLEDs using C_60_/pentacene OSHJ as charge injectors with changing either C_60_ thickness or Pentacene thickness. (a)** *J-V-L* **(b)** Current efficiency as a function of current density, and **(c)** Power efficiency as a function of current density characteristics. Device structure: ITO/C_60_(X nm)/pentacene(10 nm)/TCTA:MoO_3_(70 nm)/TCTA(10 nm)/TCTA: Ir(ppy)_2_(acac)(20 nm)/TPBi(10 nm)/TPBi:Li_2_CO_3_(40 nm)/C_60_(20 nm)/pentacene(10 nm)/Al(120 nm), X= 10, 20, 30, 40. **(d)** *J-V-L*, **(e)** Current efficiency as a function of current density, and **(f)** Power efficiency as a function of current density characteristics. Device structure: ITO/C_60_(20 nm)/pentacene(10 nm)/TCTA:MoO_3_(70 nm)/TCTA(10 nm)/TCTA: Ir(ppy)_2_(acac)(20 nm)/TPBi(10 nm)/TPBi:Li_2_CO_3_(40 nm)/C_60_(20 nm)/pentacene(X nm)/Al(120 nm), X=5, 10, 15, 25.


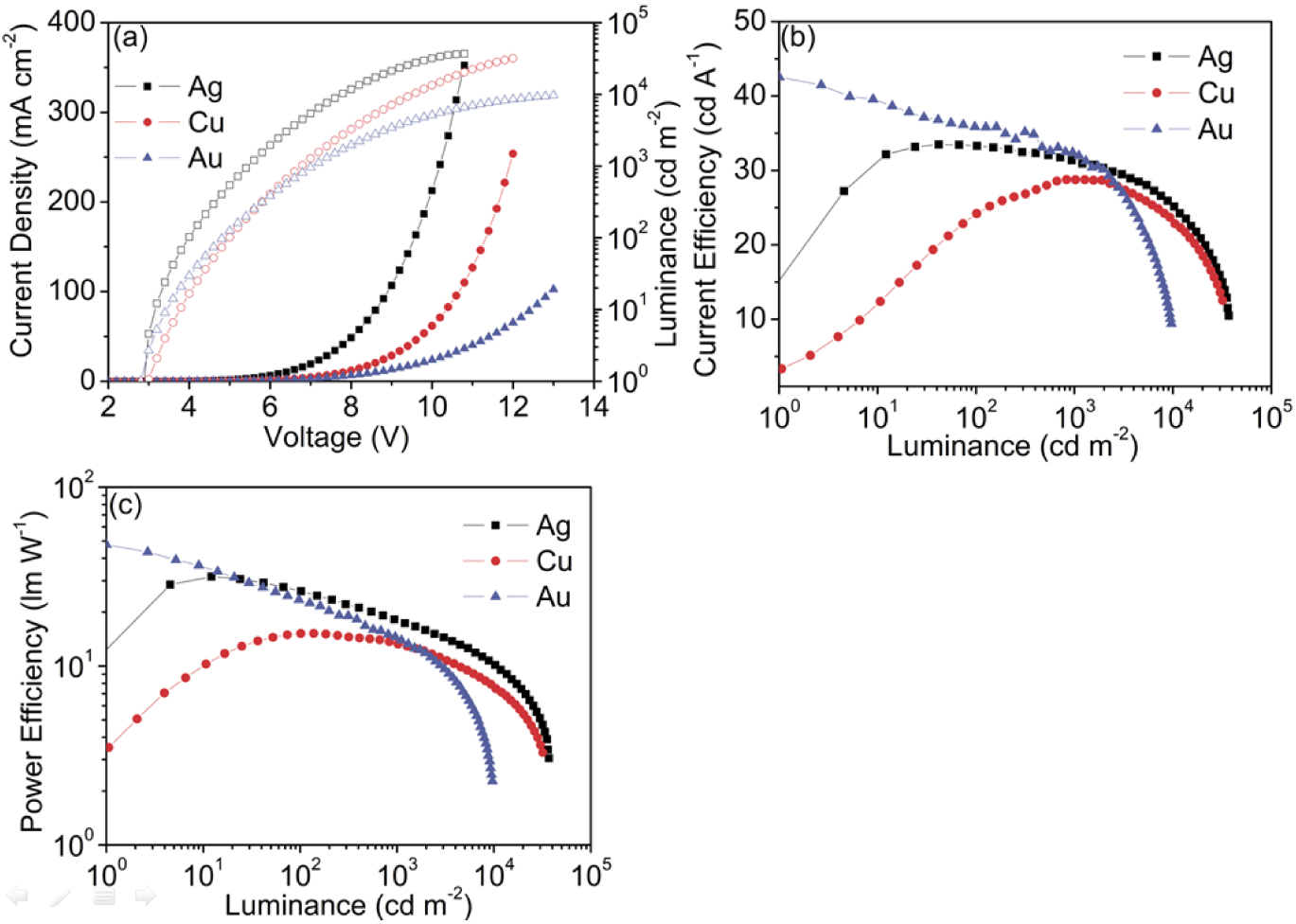


**Figure S4.** **The EL performance of the OLEDs using the different metal electrodes without the CGLs. (a)** *J-V-L*, **(b)** Current efficiency as a function of luminance, and **(c)** Power efficiency as a function of luminance. Device structure: ITO/TCTA:MoO_3_(70 nm)/TCTA(10 nm)/TCTA: Ir(ppy)_2_(acac)(20 nm)/TPBi(10 nm)/TPBi:Li_2_CO_3_(40 nm)/Ag, Cu, Au(120 nm).


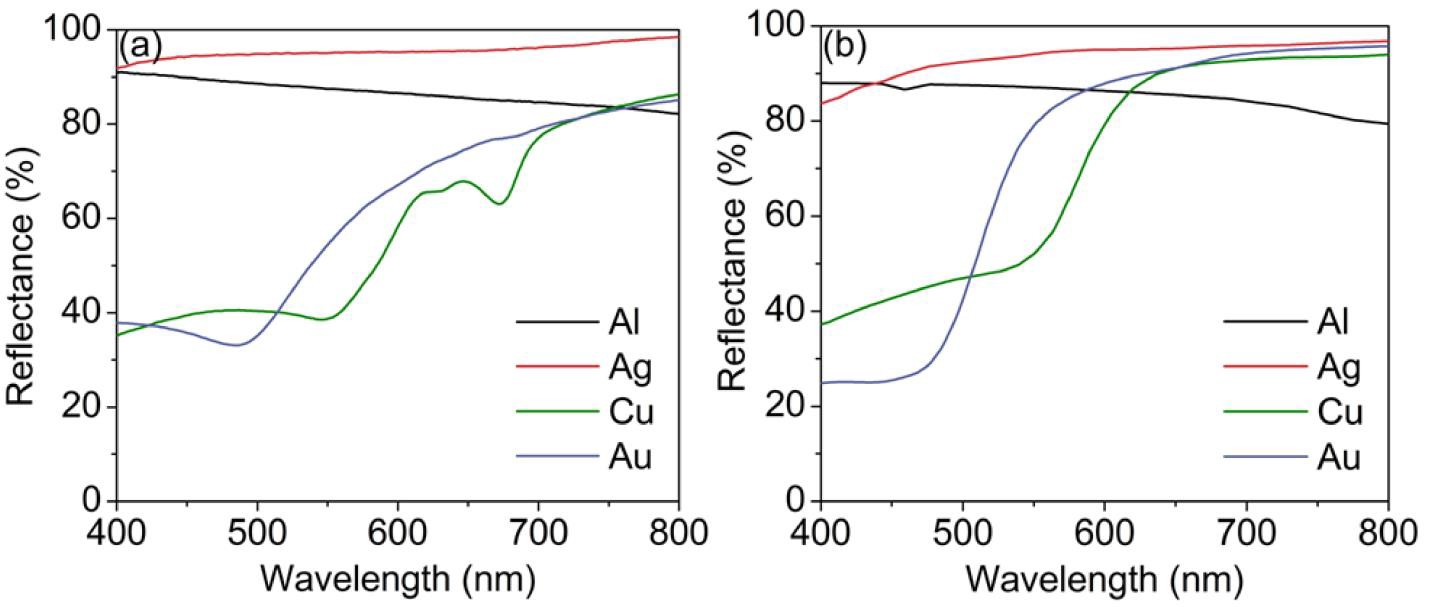


**Figure S5. The reflectance spectra of Al, Ag, Cu, and Au with the thickness of 120 nm. (a)**

Experiment and **(b)** Simulation.


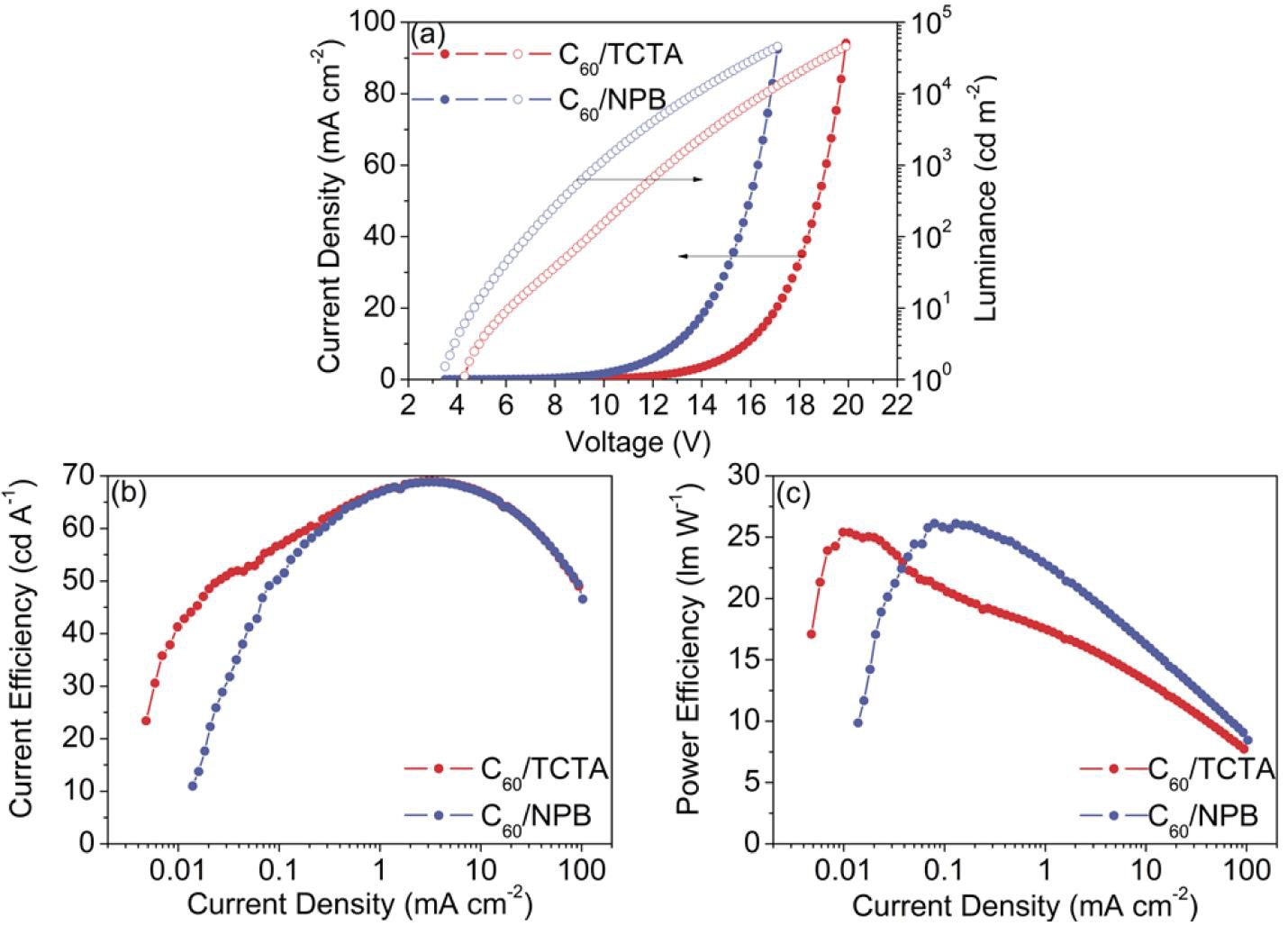


**Figure S6. Electroluminescent performances of C60/TCTA and C60/NPB OSHJs-based devices. (a)** Current density-voltage-luminance characteristics. **(b)** Current efficiency as a function of current density. **(c)** Power efficiency as a function of current density.


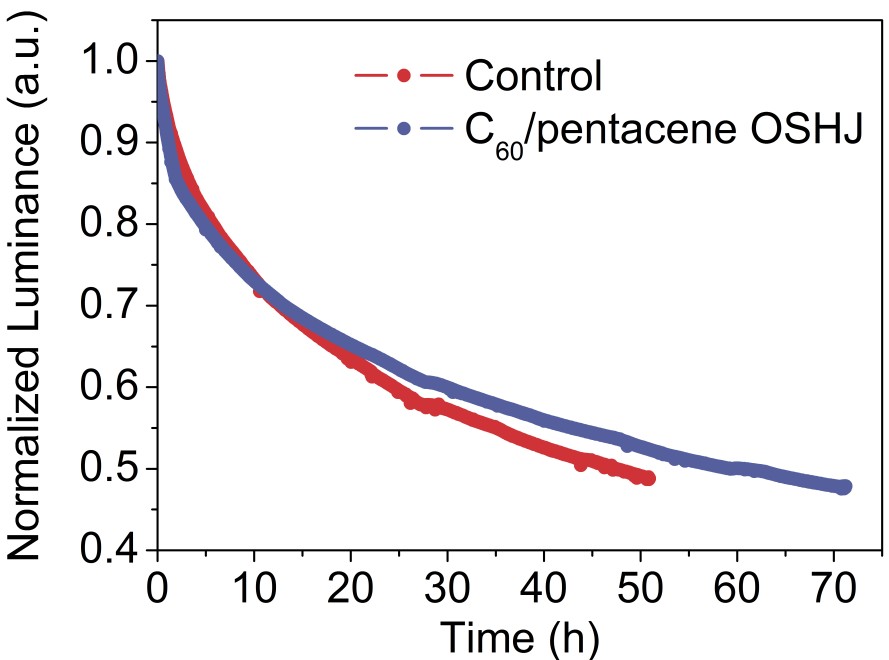


## Figure S7. Comparison of lifetime between control device and OSHJ-based device.
